# Comparative maxicircle analysis in *Trypanosoma* species from the LSRM clade highlights patterns in an underexplored lineage

**DOI:** 10.1371/journal.pone.0332749

**Published:** 2025-09-22

**Authors:** Fanny Rusman, Valeria Aramayo, Noelia Floridia-Yapur, Anahí Guadalupe Díaz, Tatiana Ponce, Soledad Hodi, Juan José Aguirre, Gonzalo Greif, Luisa Berná, Carlos Robello, Patricio Diosque, Nicolás Tomasini

**Affiliations:** 1 Instituto de Patología Experimental - Dr. Miguel Ángel Basombrío IPE-CONICET, Salta, Argentina; 2 Laboratorio de Interacciones Hospedero-Patógeno - UBM, Institut Pasteur de Montevideo, Montevideo, Uruguay,; 3 Unidad de Bioinformática, Institut Pasteur de Montevideo, Montevideo, Uruguay; University of Ostrava: Ostravska univerzita, CZECHIA

## Abstract

*Trypanosoma lainsoni, Trypanosoma platydactyli, and Trypanosoma scelopori* are kinetoplastid flagellates belonging to the LSRM clade of the genus *Trypanosoma.* These parasites have a unique mitochondrial DNA structure, the kinetoplast, comprising catenated maxicircles and minicircles. However, genetic information on the kinetoplasts of these species remains unknown. In this study, we assembled and analyzed maxicircles from different isolates of *T. lainsoni*, *T. platydactyli*, and *T. scelopori* to address the current gap in genetic information about the LSRM clade and explore their phylogenetic relationships within the *Trypanosoma* genus. The maxicircle of *T. lainsoni* isolate Le29, including the coding and divergent regions, was *de novo* assembled combining Illumina and Oxford Nanopore Technologies, revealing a length of 49,306 bp. Additional isolates of *T. lainsoni* (Ca37 and Ca47), as well as *T. platydactyli* and *T. scelopori,* were sequenced with Illumina, yielding complete coding regions and partial divergent regions for all. As in other trypanosomes, coding regions exhibited conserved synteny in gene order and RNA editing patterns. We found that mRNA editing by U-insertion was higher in *T. lainsoni* than in *T. cruzi,* suggesting a partial loss of editing in the latter. Phylogenetic analyses based on the coding region positioned the LSRM clade alongside the Aquatic clade as one of the most ancestral groups within non-salivarian trypanosomes and supported the ancestral position of the *Trypanosoma brucei* clade, contrasting with previous reports. Finally, analysis of the maxicircle divergent region suggests a gradual transition from long to short repeat structures in non-salivarian trypanosomes. This study provides the first characterization of the *T. lainsoni* maxicircle and related LSRM clade species. These findings provide new insights into the ancestral relationships within the group, the evolution of the divergent region of the maxicircles and propose RNA editing has been evolving within the genus.

## Introduction

Trypanosomes constitute a diverse group of protozoan parasites found within the genus *Trypanosoma* belonging to the higher class Kinetoplastida [1,2]. Trypanosomes conform a monophyletic clade composed by species able to infect all classes of vertebrate hosts, exhibiting various morphological forms throughout their life cycle, and being transmitted by blood-sucking invertebrates [3,4]. Some studies have explored the intricate relationships among members of the *Trypanosoma* genus [5,6]. The most taxon-rich phylogenetic trees were constructed based on alignments of 18S rDNA and gGAPDH genes and have shed light on certain relationships within the genus, delineating almost ten well-supported clades within it [7–10]. Despite these advances, controversies persist regarding the evolutionary relationships among major clades, particularly the placement of the Aquatic clade and its relation to terrestrial lineages. The Aquatic clade includes *Trypanosoma* species that infect aquatic vertebrates, such as fish, amphibians, turtles, and platypus. These species are typically transmitted by leeches and sand flies [7–10]. Some studies suggest the Aquatic clade occupies a basal position within the genus, supported by specific phylogenetic reconstructions [11–14]. However, this interpretation is controversial as other analyses using alternative methods suggest its position is not consistently resolved across datasets [10]. Similarly, the placement of the *T. brucei* clade remains a subject of debate. While some phylogenetic studies position it as an ancestral lineage within the genus [10], contrasting evidence supports its classification as a more derived group, reflecting specific evolutionary adaptations [4,12,14–16]. Furthermore, the *Trypanosoma pestanai* and LSRM (lizard-snake-rodent-marsupial) clades illustrate some of the ongoing challenges in resolving phylogenetic relationships, as their precise evolutionary positions relative to other terrestrial lineages remain controversial [10,17,18]. Despite these efforts, a clear understanding of the evolutionary relationships between different clades remains elusive, highlighting the need for alternative genetic markers, to clarify these complex phylogenetic patterns. However, by correlating the hosts of trypanosomatids with phylogenetic trees, significant associations were uncovered between clades and specific hosts [11].

Among the main clades of trypanosomes, members of the LSRM clade are distinguished by their close association with their hosts [11]. This clade includes species such as *Trypanosoma platydactyli* that has been identified in lizards, such as geckoes (*Tarentola mauritanica*) in Italy and France [19] and *Trypanosoma scelopori* that was originally described from the North American western fence lizard (*Sceloporus occidentalis*) [20]. It is important to note that these clade-host associations are not exclusive and may change as new studies emerge, as demonstrated by the discovery of *Trypanosoma lainsoni*, which was first isolated from a rodent (*Mesomys hispidus*) in the Amazon Forest (Amazonas state, Brazil) [21]. Its geographical range was later extended to the Argentine Chaco (Parque Nacional Copo), and its host record also expanded, as it was isolated for the first time from a feline (*Leopardus geoffroyi*) [18]. Recently, the list of non-volant small mammals infected by *T. lainsoni* in Brazil (Minas Gerais state, Brazil) was expanded [22]. While various characteristics of *T. lainsoni* have been described, critical aspects such as its vector(s), life cycle, pathogenicity, and potential infection of other hosts or reservoirs remain elusive.

Trypanosomes, like other kinetoplastids, exhibit distinctive features such as a unique mitochondrial DNA structure known as the kinetoplast (kDNA). The kDNA is composed of two classes of circular DNA molecules that are concatenated: minicircles and maxicircles [23–26]. Minicircles exhibit sizes ranging from 0.33 to 10 kb depending on species and are found in tens of thousands of copies per network [23–25,27,28]. Meanwhile, maxicircles which are equivalent to the mitochondrial genome of other eukaryotes exhibit species-specific sizes ranging from 20 to 64 kb and are found in dozens of copies per network [26,29–33]. Maxicircles are characterized by a coding region (CR) that encodes components of the respiratory chain, and a divergent region (DR) composed of a short repetitive region and a long repetitive region, both of unknown functions [6,29–32,34–36]. This region is challenging to sequence and assemble due to its repetitive nature and length variability across different species [6,29,37]. Although comparative studies have identified common patterns in the organization of the DR, its functional role remains largely unknown [34]. Whereas the DR has been proposed as a regulator of gene expression, or a potential replication origin, topoisomerase II binding sites, and transcripts of varying lengths within this genomic region suggests a more intricate functional role than previously hypothesized [38]. Despite these findings, the evolution of the maxicircle remains elusive. The role of the DR and its repetitive nature are not fully elucidated, and most CR genes require extensive RNA editing for functionality. The origin and diversification of this process within *Trypanosoma* remain largely unexplored, as do the evolutionary pressures shaping the DR. Clarifying these aspects is key to understanding the evolution of mitochondrial features in the genus.

The CR of the maxicircle is composed of several genes, most of them are typical protein-coding genes [30,39,40]. Of the eighteen protein-coding genes in the maxicircles, twelve are encoded as cryptogenes and lack functional open reading frames [41]. These transcripts become functional only after post-transcriptional processing—specifically, RNA editing—which involves the insertion and deletion of uridine residues [41–43]. The process is guided by gRNAs mostly encoded by the minicircles [44–47].

Here we provide the first characterization of maxicircle sequences within the LSRM clade. Using a hybrid sequencing approach, we successfully assembled and annotated the maxicircle of *T. lainsoni*. In addition, we retrieved raw sequencing data from public repositories and performed *de novo* assembly and annotation of the maxicircles of *T. platydactyli* and *T. scelopori*, which had not been previously analyzed despite the availability of their genomic datasets [48]. Phylogenetic analyses placed the LSRM clade as one of the most ancestral lineages among non-salivarian trypanosomes, confirming its basal position alongside the Aquatic clade. These findings also support the ancestral position of the *T. brucei* clade and provide new insights into evolutionary dynamics of maxicircles and RNA editing within the *Trypanosoma* genus.

## Materials and methods

### *Trypanosoma lainsoni* isolates

For this study, three isolates of *T. lainsoni* were analyzed, obtained from different hosts, all sourced from Parque Nacional Copo, Argentina. The three isolates were previously identified as *T. lainsoni* in a prior study through the amplification of 18S and gGAPDH genes [18]. Isolate Le29 was collected from *Leopardus geoffroyi*, while isolates Ca37 and Ca47 were obtained from *Calomys sp.* The isolates were cultivated in a biphasic medium, comprising a solid phase (4% agar supplemented with rabbit blood) and liver infusion-tryptose (LIT) medium enriched with 20% fetal bovine serum, hemin (20 µg/mL), penicillin (100 IU), and streptomycin (100 µg/mL). Cultures were maintained under agitation at 25°C. Parasites in the exponential growth phase were harvested through centrifugation (800 xg, 10 min, 4°C) for subsequent DNA extraction. Genomic DNA was extracted using a commercial kit (Quick-DNA MiniPrep, Zymo) following the manufacturer instructions. DNA quality and quantity were assessed by measuring the absorbance ratio at 260/280 nm and the absorbance at 260 nm using Nanodrop (Thermo Fisher Scientific, USA). Genomic DNA integrity was confirmed through electrophoresis on a 0.8% agarose gel.

### Deep sequencing

The whole genome libraries of *T. lainsoni* isolates Le29, Ca37 and Ca47 were sequenced on an Illumina NovaSeq (Illumina, San Diego, USA) with a 150 bp paired-end read approach performed by a sequencing service (Novogene, USA), obtaining a total of 41 million paired-end reads for Le29, 35 million for Ca37, and 43 million for Ca47. Additionally, a whole genome library was prepared for Le29 with the SQK-NBD112–24 Kit (Oxford Nanopore Technologies, UK) according to the manufacturer instructions and sequenced using a MinION Mk1C platform (Oxford Nanopore Technologies, UK). The library was run for 48 hours in a R10.4 FlowCell (FLO-MIN112, Oxford Nanopore Technologies, UK) starting from 1 μg of total genomic DNA, yielding a total of 45,397 reads. Basecalling of ONT reads was performed using Guppy v6.1.5 (Oxford Nanopore Technologies, UK) in high-accuracy mode, executed through MinKNOW v22.05.8.

### Preprocessing reads

The analyses were carried out on Galaxy (https://nanopore.usegalaxy.eu/). The first step involved quality control for both ONT and Illumina reads using different strategies. Long reads (ONT) were assessed using NanoStat [49], while Illumina reads were evaluated using FastQC [50] and Falco [51]. Illumina reads for *T. lainsoni* isolates, *T. platydactyli* (SRR24223604), and *T. scelopori* (SRR24223650) were trimmed and quality-filtered using Trimmomatic (version 0.38.01) [52] with the following parameters: LEADING: 3 TRAILING: 3 SLIDINGWINDOW: 4:20 MINLEN: 50.

### Maxicircle assembly and gene annotation

A *de novo* hybrid assembly was generated using MaSuRCA (v4.1.4) [53], combining ONT long reads and Illumina paired-end short reads from the Le29 isolate, with the specific aim of recovering the mitochondrial maxicircle. To identify the maxicircle within the assembly, BLASTn searches were performed using known maxicircle sequences (S1 Table) as queries. This search yielded a single contig with high similarity to reference maxicircles, hereafter referred to as the maxicircle-like contig. This contig was manually reordered to place the 12S rDNA gene at the starting coordinate, in accordance with the orientation commonly used for maxicircle sequence reports. The reordered sequence was then aligned with representative maxicircle sequences (S1 Table) using MEGA7.0 [54] to assess gene collinearity and support subsequent annotation and comparative analyses. Subsequently, a two-step polishing strategy was applied. First, ONT reads were mapped with minimap2 (v2.28) [55] to the reordered contig, which served as the reference for Medaka (v1.7.2) (https://github.com/nanoporetech/medaka) polishing using the appropriate model for R10.4.1 chemistry and high-accuracy basecalling. Second, quality-trimmed Illumina reads were mapped with BWA-MEM2 (v2.2.1) [56] to the Medaka-polished contig, which served as the reference for Pilon (v1.20.1) [57] to correct any residual base-level errors. The final maxicircle sequence obtained after this step was used in all downstream analyses. To validate the integrity and continuity of the conserved region (CR) obtained from the hybrid assembly, we also performed an independent *de novo* assembly using NOVOPlasty (see below). This independent reconstruction served as confirmation of the structure recovered by the hybrid strategy. Finally, to assess sequencing depth and assembly accuracy, ONT and quality-filtered Illumina reads were independently mapped to the polished maxicircle sequence using minimap2 (v2.28) and BWA-MEM2 (v2.2.1), respectively, using default parameters. The resulting BAM files were sorted with Samtools sort (v2.0.7) [58] and duplicate reads were marked using Picard MarkDuplicates (v3.1.1.0). Per-base depth and overall coverage were calculated with Samtools (v1.21) [58]. Assembly consensus quality (QV) was estimated from mismatch and indel statistics obtained with Qualimap BamQC (v2.3.0) [59] for the entire maxicircle, and in particular for the coding region, and coverage profiles were visualized with Matplotlib [60].

Annotation of maxicircle genes was performed manually based on multiple sequence alignments (MSA) generated with MEGA7.0. Our assembled maxicircle sequences were aligned to annotated maxicircle references from *T. cruzi*- Esmeraldo (DQ343646.1), *T. marinkellei* (KC427240.1), *T. cruzi*- Sylvio (FJ20396.1), *T. equiperdum* (EU185800.1), *T. vivax* (KM386508.1), *T. musculi* (KT368148.1), *T. lewisi* (OM000219.1), *T. copemani* (MG948557.1), and *L. tarentolae* (NC000894.1). Gene boundaries (start and stop codons) for non-edited genes were determined based on conserved synteny and ORFs that translated into coherent protein sequences.

For extensively edited genes, inferred mature mRNA sequences were used to define gene limits and validate annotation. The RNA editing patterns of *T. lainsoni* maxicircle genes were inferred by manually reconstructing edited mRNA sequences for each gene, using the editing profiles of orthologous genes in *T. cruzi* as reference. Uridine insertions and deletions were introduced into unedited sequences to generate functional open reading frames, following the approach described previously in [40,61–63]. The total number of U-indels per gene was recorded to estimate the extent of editing. GC content was calculated separately for each gene and used only as a complementary metric to describe GC variation across genes, since pan-edited genes have been associated with elevated GC content in previous studies [30,36,64], without being used to infer editing. The GC percentage and the GC skew were analyzed at a 50 bp window and visually represented with the annotated maxicircle genome using pyCirclize (https://github.com/moshi4/pyCirclize). Additionally, AT percentage and AT skew were calculated for the complete maxicircle sequences of Le29 and *T. cruzi*-Dm28c using a 100 bp sliding window, to address the existence of an AT-rich region. The Dm28c sequence was selected for comparison because it represents a fully assembled maxicircle, including both the conserved and divergent regions, and has been previously reported to contain an AT-rich region [29].

A dot plot graph illustrating the self-alignment of the *T. lainsoni* maxicircle sequence was generated using YASS webserver (https://bioinfo.univ-lille.fr/yass/yass.php) with default parameters. Additionally, dot plot graphs were created to compare the *T. lainsoni* maxicircle CR sequence with that of five other Trypanosomatidae species.

### Assembly of the maxicircle coding region of *Trypanosoma lainsoni* isolates Ca37 and Ca47, *Trypanosoma platydactyli* and *Trypanosoma scelopori*

For *T. lainsoni* isolates Ca37 and Ca47, *T. platydactyli* (NCBI-SRA: SRR24223604) and *T. scelopori* (NCBI-SRA: SRR24223650) [48] the respective coding regions of the maxicircle were assembled using NOVOPlasty (version 4.3.1) [65]. The parameters employed were as follows: the trimmed paired-end reads (R1 and R2), generated by Trimmomatic, were used as input for the assembly. The seed sequence used for assembly was obtained from the *T. cruzi* (Sylvio) maxicircle sequence (NCBI code: FJ203996.1), utilizing the non-edited gene cytochrome oxidase subunit 1 (COI) as the initial point for assembly. Other parameters were configured as follows: The Illumina platform was specified with a read length of 150 bases and a total insert size of 300 bases for paired-end reads. The assembly parameters were designated for mitochondrial assembly, anticipating a genome size range of 12,000–52,000 bases. It is worth noting that the program reconstructs the complete CR along with DR sequences flanking it. However, due to the highly repetitive nature of the DR, these sequences are collapsed by the program, preventing the resolution of its full extent.

To assess structural conservation, dot plot graphs using YASS with default parameters were generated to compare the CR of *T. lainsoni* (Le29, obtained from the hybrid assembly) with those obtained for Ca47, Ca37, *T. platydactyli* and *T. scelopori*. Additionally, self-alignments of Le29 and Ca47 (NOVOPlasty obtained contig) were performed to explore internal repetition patterns within the DR, particularly at its junction with the CR.

### Divergent region analyses

Dot plot graphs were generated using YASS webserver with default parameters to compare the complete *T. lainsoni* maxicircle sequence, including its divergent region, with those of *T. cruzi* (MW421590.1), *T. musculi* (OM000218.1), *T. brucei brucei* (M94286.1), *T. lewisi* (OM000219.1), and *T. vivax* (MT090068.1). Outputs from the YASS webserver were used to identify palindromic sequences within the DR of *T. lainsoni*. Upon completing the search, palindromic sequences were extracted from the YASS alignment files. Subsequently, the nucleotide sequences were aligned using MEGA7.0.

### Phylogenetic analysis

The maxicircle coding regions of LSRM clade species were compared with the available trypanosome maxicircles sequences in Nucleotide (NCBI) (S1 Table). The entire CR of the maxicircles were aligned and trimmed using MEGA7.0. Phylogenetic analysis was performed by the maximum likelihood (ML) method, using MEGA7.0. The best substitution model was selected using the AIC criterion. The ML tree was generated with 1,000 bootstrap replicates. In addition, the tree was edited using FigTree (version 1.4.4) (http://tree.bio.ed.ac.uk/software/figtree/).

## Results

### Assembly and annotation of kDNA maxicircles in the LSRM clade

**Fig 1:**
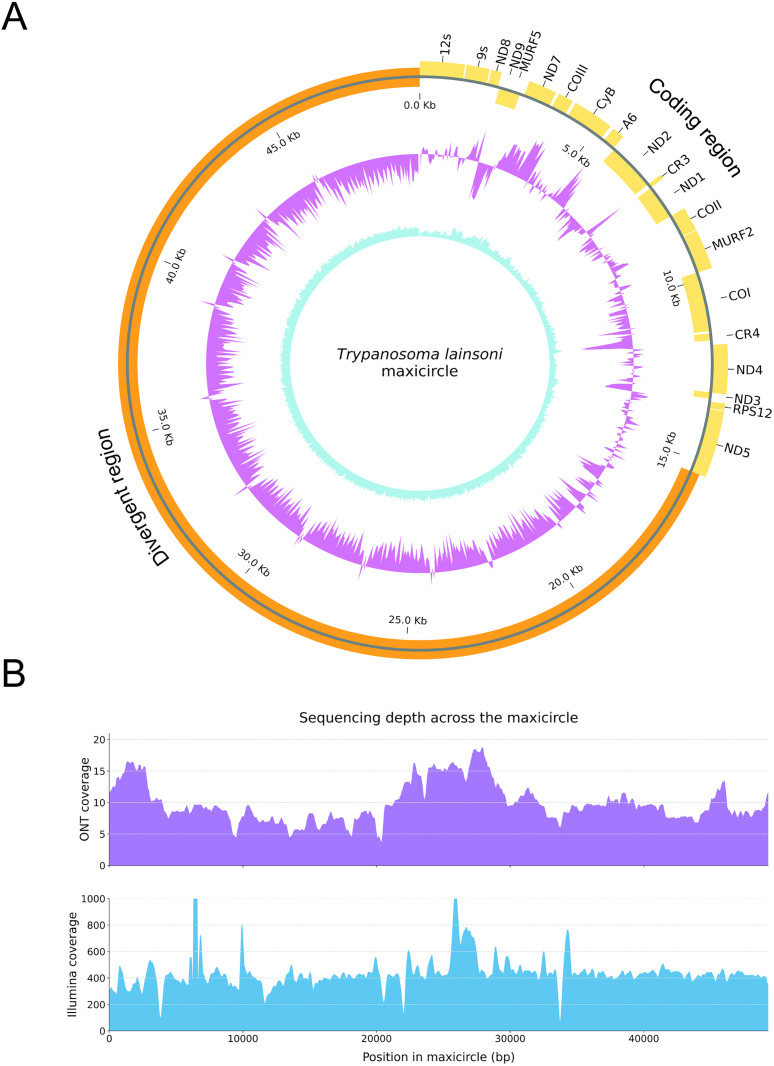
*Trypanosoma lainsoni* (Le29) maxicircle schematic representation. **(A)** Annotated genes are displayed in the outer circle in light orange (outer + strand and inner – strand) and the divergent region in orange. The GC skew with a window size of 50 bp is illustrated in violet. The GC content (also with a window size of 50 bp) is illustrated in light blue. **(B)** Depth of coverage was calculated for each position along the maxicircle using long-read (Nanopore; upper panel) and short-read (Illumina; lower panel) sequencing data. Nanopore reads were aligned using minimap2 and Illumina reads using BWA-MEM2. The x-axis represents the nucleotide positions along the maxicircle (in base pairs) and the y-axis indicates the number of reads mapped to each position (read depth) calculated using Samtools and plotted with Matplotlib. Each panel shows the coverage profile corresponding to the sequencing platform.

Genomic DNA from *T. lainsoni* Le29 isolate was sequenced using both the Illumina and ONT platforms to obtain the maxicircle sequence. A *de novo* hybrid assembly was generated with MaSuRCA using the complete set of reads from both platforms. The maxicircle was identified by performing BLASTn searches against the hybrid MaSuRCA assembly contigs, using available maxicircle sequences of other *Trypanosoma* species as queries (Supplementary File 3). This approach allowed the detection of a 49,255 bp maxicircle-like contig containing the complete coding region flanked by the divergent region. The maxicircle-like contig was manually reordered at the 12S gene. The sequence was then polished in two steps: first with Medaka using ONT long reads, and subsequently with Pilon using quality-filtered Illumina short reads. The final curated *T. lainsoni* maxicircle sequence was 49,306 bp in length, comprising a 15,340 bp coding region and a 33,966 bp divergent region (Fig 1). Notably, the *T. lainsoni* maxicircle does not present an AT-rich region (S1 Fig). To further evaluate the structure of the CR, an independent *de novo* assembly was carried out using NOVOPlasty with Illumina reads. The resulting contig corresponded to the expected organization of the CR and showed no inconsistencies with the curated maxicircle sequence. Sequencing coverage across the ~ 49 kb maxicircle was evaluated independently for both platforms after polishing. Illumina reads provided high-quality coverage across the whole molecule (Fig 1B). ONT reads, while fewer in number, included long reads that spanned the entire structure and supported the continuity of both conserved and divergent regions. Summary statistics on read quality (S2 Fig and S3 Fig) and length distribution for both platforms are provided in S2 Table, and S3 Table. For Illumina reads mapped to the complete maxicircle, the mean coverage was ~ 472.9× with a consensus quality value (QV) of ~27.2 (~99.81% accuracy), while ONT reads achieved a mean coverage of ~10.48 × . For the coding region, Illumina reads yielded a mean coverage of ~418.6 × , supporting both the structural and nucleotide-level fidelity of the assembly.

The nucleotide sequences and gene arrangement within the CR of the maxicircle were highly conserved across trypanosomatids, while allowed for some species-specific variations. This conservation provided a framework for the annotation of 20 genes in the kDNA maxicircles from the three species (Table 1), including the start and end positions of these genes and their length comparison with *T. cruzi*. Additionally, the maxicircles from two isolates of *T. lainsoni* (Ca37 and Ca47), as well as from *T. platydactyli* and *T. scelopori*, were assembled (S1 File) and annotated. Furthermore, CR regions of these isolates were analyzed and presented in S4 Fig. These analyses revealed a high degree of sequence conservation across the CR of the LSRM clade, with remarkable preservation in gene order and overall size. However, specific differences were observed. For instance, MURF5 showed notable variation in length, being 321 bp in *T. lainsoni*, 252 bp in *T. scelopori*, and 213 bp in *T. platydactyli*, while significantly shorter in *T. cruzi* at 148 bp based on NCBI annotations. Notably, previous studies have reported a longer MURF5 sequence of 261–267 bp for *T. cruzi* [40]. Similarly, in 8 out of the 9 extensively edited genes, the length is shorter in *T. lainsoni* compared to *T. cruzi*, primarily due to a lower thymine content in *T. lainsoni*. These findings are summarized in Table 1.

**Table 1:** Comparison of gene positions and lengths among LSRM clade species and T. cruzi.

Gene	RNA editing	*T. lainsoni* Le29 (Start-End, Length)^a^	*T. lainsoni* Ca37 (Start-End, Length)	*T. lainsoni* Ca47 (Start-End, Length)	*T. platydactyli* (Start-End, Length)	*T. scelopori* (Start-End, Length)	*T. cruzi*^b^ length	Strand
**12s**	–	1-1158(1158)	576-1733(1158)	1315-2472(1158)	1172-2324(1153)	1950-3102(1153)	1159	+
**9s**	–	1198-1800(603)	1773-2376(604)	2512-3114(603)	2362-2968(607)	3151-3758(608)	610	+
**ND8**	Extensive	1844-2115(272)	2420-2691(272)	3158-3428(271)	3014-3283(270)	3803-4101(299)	280	+
**ND9**	Extensive	2169-2491(323)	2745-3067(323)	3482-3804(323)	3325-3676(352)	4143-4482(340)	350	–
**MURF5**	None	2425-2745(321)	3001-3321(321)	3738-4058(321)	3669-3881(213)	4483-4734(252)	148	–
**ND7**	Extensive	2858-3596(739)	3434-4172(739)	4171-4909(739)	4013-4764(752)	4834-5608(775)	747	+
**COIII**	Extensive	3669-4076(408)	4245-4652(408)	4982-5389(408)	4833-5252(420)	5699-6134(436)	420	+
**CyB**	Minor	4160-5239(1080)	4736-5815(1080)	5473-6552(1080)	5331-6410(1080)	6219-7298(1080)	1081	+
**A6**	Extensive	5336-5663(328)	5912-6238(327)	6649-6975(327)	6457-6791(335)	7343-7658(316)	330	+
**ND2**	None	5705-7030(1326)	6280-7605(1326)	7017-8342(1326)	6845-8170(1326)	7707-9032(1326)	1341	–
**CR3**	Extensive	7031-7142(112)	7606-7717(112)	8343-8454(112)	8170-8282(113)	9033-9152(120)	122	+
**ND1**	None	7147-8088(942)	7722-8663(942)	8459-9400(942)	8277-9218(942)	9148-10089(942)	942	–
**COII**	Minor	8092-8720(629)	8667-9295(629)	9404-10032(629)	9225-9853(629)	10093-10721(629)	629	+
**MURF2**	Minor	8743-9796(1054)	9318-10371(1054)	10055-11108(1054)	9873-10927(1055)	10741-11793(1053)	1054	+
**COI**	None	9786-11432(1647)	10361-12007(1647)	11098-12744(1647)	10917-12563(1647)	11783-13429(1647)	1650	–
**CR4**	Extensive	11493-11690(198)	12068-12265(198)	12805-13002(198)	12619-12809(191)	13484-13697(214)	209	–
**ND4**	None	11812-13125(1314)	12387-13700(1314)	13124-14437(1314)	12924-14237(1314)	13820-15133(1314)	1313	+
**ND3**	Extensive	13109-13283(175)	13684-13858(175)	14421-14595(175)	14221-14389(169)	15126-15316(191)	193	–
**RPS12**	Extensive	13366-13548(183)	13941-14123(183)	14678-14860(183)	14482-14663(182)	15395-15586(192)	192	+
**ND5**	None	13568-15340(1773)	14143-15915(1773)	14880-16652(1773)	14682-16454(1773)	15609-17381(1773)	1771	+

^a^Gene positions are depicted in reference to the start of 12S rRNA gene.

^b^*T. cruzi* strain Sylvio (NCBI: FJ203996.1).

The overall architecture of the *T. lainsoni* maxicircle, including annotated genes of the CR, DR, GC skew and content across the whole maxicircle is illustrated in Fig 1.

**Fig 2:**
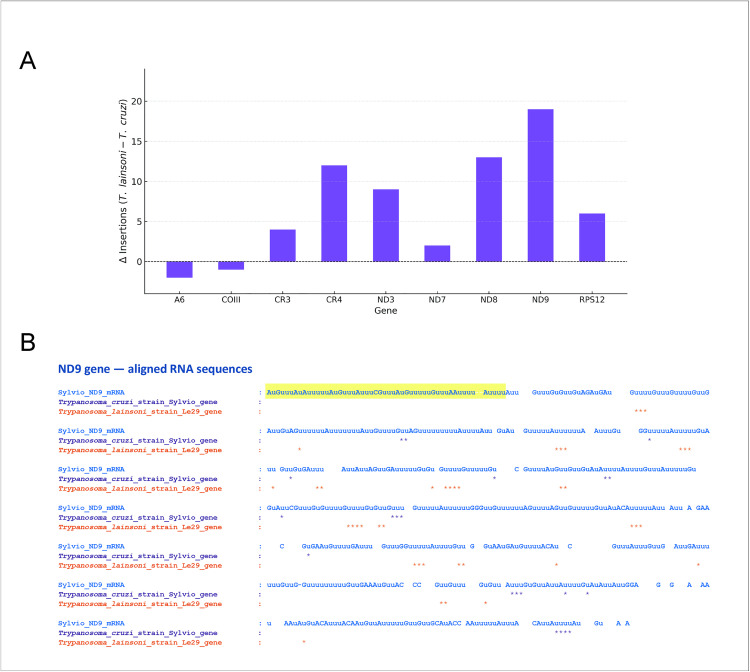
Differences in inferred insertion events between *Trypanosoma lainsoni* and *Trypanosoma cruzi.* **(A)** Bar plot showing the difference in the number of insertion events inferred from multiple sequence alignments between *T. lainsoni* Le29 and *T. cruzi* Sylvio X10 across nine mitochondrial maxicircle genes. Insertions were estimated as alignment positions where the edited transcript contains a nucleotide aligned to a gap in the genomic sequence. Values represent Δ insertions (*T. lainsoni* – *T. cruzi*). A value > 0 indicates more U-insertions in *T. lainsoni* than in *T. cruzi*. **(B)** Schematic representation of inferred U-insertion sites for the ND9 gene, based on the alignment between *T. cruzi* Sylvio X10 and *T. lainsoni* Le29. The fully edited mRNA sequence of *T. cruzi* Sylvio X10 is shown in blue (top). Asterisks indicate positions with U-insertions in the edited transcript relative to the genomic sequences: light purple for *T. cruzi* Sylvio X10 and orange for *T. lainsoni* Le29. The yellow box highlights a region that was not evaluated due to sequence differences between *T. cruzi* and *T. lainsoni*.

Eighteen protein-coding genes were identified and classified based on the extent of RNA editing required to produce functional transcripts (Table 1). To complement this annotation, GC skew analysis revealed a strong G-rich bias, particularly in genes subjected to extensive RNA editing (S5 Fig). The corresponding inferred edited mRNAs are available in S2 File. Finally, the number of insertions required to obtain the open reading frame was analyzed, revealing that in seven out of nine extensively edited genes, *T. lainsoni* requires a higher number of uridine insertions compared to *T. cruzi* (Fig 2A). A specific example is shown for the gene ND9 in Fig 2B, which illustrates the increased editing complexity in *T. lainsoni*.

### Ancestral position of *T. brucei*, LSRM and aquatic clades within *Trypanosoma*

The phylogenetic analysis of the CR revealed a robust clustering of trypanosome species into eight distinct clades within the genus *Trypanosoma*. These clades include the basal *T. brucei* clade, which predominantly comprises trypanosomes infecting African mammals; the Aquatic clade, the LSRM clade; the Crocodilian clade, including trypanosomes infecting crocodilians and alligators; the *T. theileri* clade, with species infecting marsupials and placental mammals; the *T. pestanai* clade, represented by *T. copemani*, a trypanosome infecting Australian marsupials, and *T. caninum*, which infects Brazilian dogs; the *T. lewisi* clade, predominantly found in rodents; and the *T. cruzi* clade, which includes trypanosomes infecting a variety of mammals with a global distribution.

**Fig 3:**
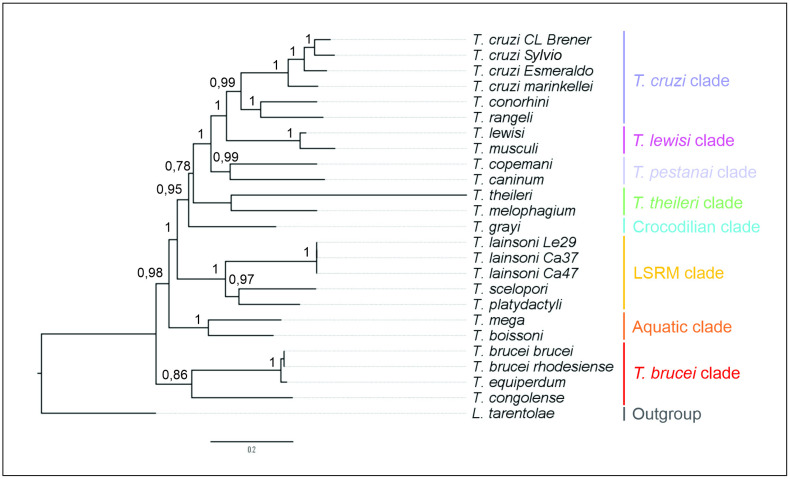
Phylogenetic analysis of the Trypanosomatidae species using the whole maxicircle coding regions. Phylogenetic tree is performed based on Maximum likelihood method based on the General Time Reversible (GTR) substitution model with discrete Gamma distribution (+G) and Invariant Sites (+ I) parameters with 1,000 bootstrap replicates with the respective bootstrap confidences indicated at branch points. The sequences used in the analysis and their corresponding GenBank accession numbers are as follows: *T. cruzi* Esmeraldo (DQ343646.1), *T. cruzi* CL Brener (DQ343645.1), *T. cruzi* Sylvio (FJ203996.1), *T. marinkellei* (KC427240.1), *T. conorhini* (MKKU01000412.1), *T. lewisi* (OM000219.1), *T. musculi* (KT368148.1), *T. copemani* (MG948557.1), *T. grayi* (OM049542.1), *T. congolense* (GCA_003013265.1), *T. brucei brucei* (MK584625.1), *Leishmania tarentolae* (NC000894.1), *T. rangeli* (KJ803830.1), *T. theileri* (GCF_002087225.1), *T. brucei rhodesiense* (OM049543.1), *T. mega* (GCA_030849715.1), *T. boissoni* (GCA_030849725.1), *T. melophagium* (GCA_022059095.1), *T. equiperdum* (CZPT020000280.1), *T. caninum* (GCA_036321205.1).

Within the phylogenetic tree, *T. brucei* clade emerges as the basal lineage, diverging first among the *Trypanosoma* species, indicating its ancestral position. Following this, two main lineages branch out: one comprising the aquatic trypanosomes, and the other including various terrestrial clades. The LSRM clade is positioned as one of the most ancestral groups alongside the aquatic clade, supported by a bootstrap value of 100%. Furthermore, isolates Le29, Ca37, and Ca47 formed a well-defined clade, indicating their close phylogenetic relationship (Fig 3).

### LSRM clade sheds light on divergent region evolution

**Fig 4:**
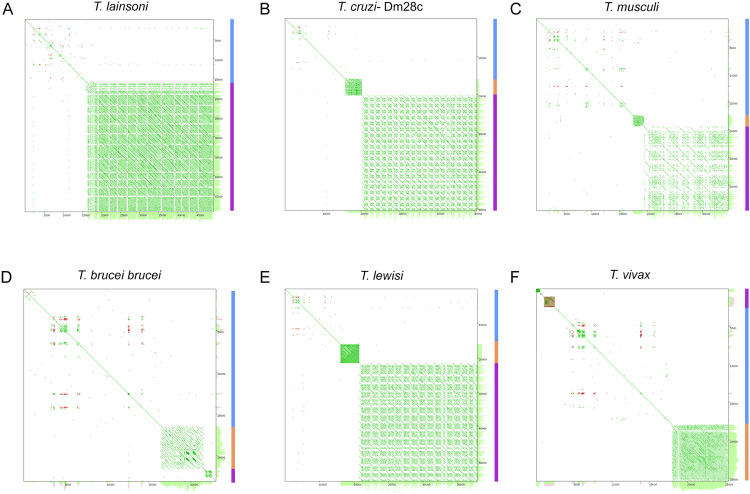
Dot plot of six whole maxicircle sequences. Dot plots were generated using YASS of self-self sequence of the six species analyzed. **(A)** T. lainsoni, **(B)** T. cruzi-Dm28c, **(C)** T. musculi, **(D)** T. brucei brucei, **(E)** T. lewisi, **(F)** T*. vivax*. The color-coded regions on the right represent key structural features of the maxicircle: blue represents the conserved region (CR). Orange and purple correspond to the divergent region, which includes the short repeats and long repeats, respectively.

Upon sequence analysis of the maxicircle DR, various tandem repeats were identified. Dot plots of maxicircles for six different trypanosomatid species, including *T. lainsoni*, are shown in Fig 4. The plots show variants of DR organization, highlighting the unique features across species. Architectural features of the DR sequence revealed several common traits: repetitive elements were consistently arranged in a head-to-tail manner, and within DR sequences, a short repeat element (also known as P5 or DRI) and a long repeat (also named P12 or DRII) could be identified. These regions consist of repeated sequences characterized by distinct differences in repeat lengths, structures, and repetition patterns. The short repeat constitutes a tandem repeat with a large period but a limited number of repetitions. However, as shown in Fig 4A, *T. lainsoni* lacks a clearly differentiated short repeat region, unlike *T. cruzi* or *T. musculi,* or *T. lewisi* (Fig 4B, C, E).

**Fig 5:**
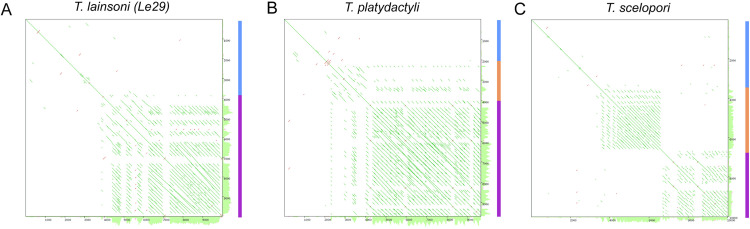
Dot plots of the CR-DR boundary in maxicircles from the LSRM clade. Dot plots were generated using YASS to compare the self-self sequences of the analyzed LSRM species, allowing the visualization of both conserved and divergent regions. **(A)** T. lainsoni (Le29), **(B)** T. platydactyli, **(C)** T*. scelopori*. The color-coded regions on the right represent key structural features of the maxicircle: blue represents the conserved region (CR). In A, a magnified view of the 5’ start of the divergent region is shown to highlight the internal repeat structure. The sparser pattern in this plot reflects the zoomed-in scale and the complex organization of repeats. Orange and purple correspond to the divergent region, which includes the short repeats and long repeats, respectively.

In contrast, a long repeat comprises highly repetitive units with a small period and is organized into repeat arrays of variable length. This organization was also observed in assemblies generated independently with NOVOPlasty for Le29 and Ca47 (S6 Fig), confirming the presence of tandem arrays with short repeat units in multiple *T. lainsoni* isolates. The YASS dot plot analysis of *T. platydactyli* and *T. scelopori* reveals an apparent progressive reduction in sequence identity within the long repeat array in relation to *T. lainsoni* (Fig 5). In *T. platydactyli*, an emerging short repeat is observed with some regions that maintain some degree of sequence identity with elements of the long repeat. Instead, in *T. scelopori*, a clearly differentiated short repetitive region becomes evident. Considering the basal position of the LSRM in relation to other terrestrial trypanosomes in the phylogenetic tree (Fig 3) with a short repeat region, these findings indicate that the short repetitive region originated from a unit of the long repeat array through the gradual loss of sequence identity and the posterior period increase.

The arrays in the long repeat region are frequently interspersed with repeat units of a different type, known as I-elements. These elements were originally described as conserved palindromic sequences in the variable region of the *T. cruzi* maxicircle [36], and later formalized as “I-elements” in comparative studies of kinetoplastid, where they mark the boundaries of repeat arrays within the divergent region. These I-elements influence the nature of large repeats; however, in some species, this element is absent (Fig 4D) [34]. For *T. lainsoni* the repetitive element I, had a length of about 390 bp that remarks the arrays borders (S7 Fig A). The cross-structure (S7 Fig B) present within the conserved element is due to a match to the reverse complement that forms an imperfect palindrome. Three types of palindromes were detected, differing in length, location, and number of occurrences relative to element I (S7 Fig B). Palindrome type I (53 bp) was located at the first occurrence of element I in the divergent region. Palindrome type II (39 bp) was found in the second repetition of element I. Finally, palindrome type III (65 bp) was observed in the remaining repetitions (S7 Fig C). Despite their varying lengths and sequences, palindromes share a 39 bp core that contains a highly conserved A_5_C element (S7 Fig D).

## Discussion

Here, we successfully assembled the *T. lainsoni* Le29 maxicircle through a *de novo* hybrid assembly combining long-read ONT and short-read Illumina sequences. Additionally, partial maxicircle sequences were assembled for two other *T. lainsoni* isolates, as well as for *T. platydactyli*, and *T. scelopori*. To the best of our knowledge, these represent the first descriptions of maxicircle sequences for these species and within the LSRM clade. We also analyzed intraspecific diversity and phylogenetic relationships with other trypanosomes, providing insights into the evolutionary relationships among clades within the genus *Trypanosoma* and the evolution of the divergent region of maxicircles. In addition, these sequences will help to better describe *T. lainsoni* distribution and host range by designing PCR specific primers to directly detect it on samples without requiring sequencing such as previous molecular methods that identified this parasite.

The estimated size of the maxicircle is 49 kb in the Le29 isolate, comprising two distinct regions: a CR of 15 kb and a DR of 33 kb (Fig 1). The maxicircle assembled with MaSuRCA for Le29 encompassed an almost complete sequence and is close to sizes reported for maxicircles of other trypanosomatid species (∼20–64 kb) [26], in particular those reported for some *Trypanosoma cruzi* strains [27,29]. The robustness of the final maxicircle sequence was supported by sequential polishing with ONT and Illumina reads, which corrected residual errors and reinforced confidence in the structural and nucleotide-level accuracy of the assembly. This is further substantiated by the high mean coverage (~472.9×) and a consensus quality value (QV) of ~27.2 (~99.81% accuracy) from read-to-assembly alignments, indicating both depth and quality sufficient to ensure reliable reconstruction. In addition, to further support the structural accuracy of the conserved region, a separate *de novo* assembly was performed using NOVOPlasty, confirming consistency in gene order within this region (Supplementary File 1). The primary size variation between the *T. lainsoni* maxicircle described in this study and those of other kinetoplastids can be attributed to differences within the DR.

Notably, the size of the CR and gene order is similar to those reported for *T. brucei*, *T. cruzi* and *T. lewisi*, which are approximately 15 kb in length [27,29,30,33,37]. Interestingly, extensively edited genes ATPase6, ND9, ND8, ND7, COIII, CR3, CR4, ND3 and RPS12 showed variations in size when compared to genes reported for *T. cruzi* (Table 1). It is predicted that these size discrepancies will be corrected through the insertion and deletion of uridine residues in the primary transcripts via the process of RNA editing. However, it is notable that in 8 of the 9 extensively edited genes, the size is smaller in *T. lainsoni* than in *T. cruzi*. These size differences are primarily due to a lower T content in *T. lainsoni*. In addition, more U-insertions were predicted for seven out of nine extensively edited genes in *T. lainsoni* than in *T. cruzi* to obtain the complete open reading frame. As *T. lainsoni* is positioned more basally than *T. cruzi* in the trypanosome phylogenetic tree, this finding supports the hypothesis that RNA editing is being partially lost during the evolution of trypanosomes, likely through a mechanism distinct from the retroposition of already edited mRNAs [66,67]. In relation to these findings, a study by [64] showed that the maxicircles of salivarian trypanosomes have high GC content and that pan-edited genes are shorter, thus requiring greater RNA editing than non-salivarian trypanosomes. However, while [64] associates these patterns with the recent evolutionary divergence in Salivarian and proposed that mRNA editing is not always “on the way out”, our results suggests a contrasting perspective: mRNA editing may be undergoing a partial reduction during the evolution of *Trypanosoma*.

In this regard, the CR sequence has proven to be a valuable taxonomic marker for phylogenetic analyses [68,69], allowing us to better resolve the evolutionary relationships between the LSRM, *T. brucei* clade and other trypanosomes. Our analysis built a robust phylogeny with high bootstrap support, effectively resolving major clades within *Trypanosoma*. Notably, the phylogenetic analyses positioned the *T. brucei* clade as basal and the LSRM clade alongside the Aquatic clade as one of the most ancestral groups within non-salivarian trypanosomes (Fig 3). This finding highlights the value of using maxicircle CR sequences to clarify evolutionary relationships that remain ambiguous with other currently used markers, offering a faster mutation rate and providing a greater number of phylogenetically informative sites [64,70]. While our findings contribute to a more comprehensive understanding of *Trypanosoma* clades relationships, further research including more species is needed to finally confirm relationships among clades.

Lastly, the discussion surrounding the DR of the kinetoplast maxicircle has long been debated. Initially characterized as a variable and non-coding region, the structural diversity observed in the DR across different species added complexity to its nature. The distinct differences in DR structures among various species have led to questions about its potential functional significance. Our analyses uncovered a 33,966 bp repetitive region comprising the DR of the *T. lainsoni* maxicircle, which lacks an AT-rich region or a clearly differentiated short repeat region (S1 FigA), as reported in other trypanosomes such as *T. cruzi* [29]. Instead, *T. lainsoni* appears to have one of the elements from the long repeat array with regions where it has lost sequence identity with other repeats. Notably, this architecture was consistently recovered not only in the hybrid assembly, but also in the ONT long reads themselves and in independent reconstructions using NOVOPlasty for both Le29 and Ca47, supporting the biological reality and conservation of this structure across isolates (Supplementary File 1, and S6 Fig). This loss of sequence identity is more evident in *T. platydactyli*, showing the emergence of a short repetitive region and, ultimately, a well-differentiated short repetitive region in *T. scelopori*. In addition, the short repeat region in *T. scelopori* has short and long period repeats (only long period repeats are observed in *T. cruzi* and others), which suggest an increase in the repetition period, possibly by reparation based on homology or slippage during replication. These results suggest a potential mechanism for the origin of the short repetitive region from the long repeat arrays in trypanosomes. A recent study by Gerasimov et al. [34] proposed that the divergent region of the maxicircle is organized into two distinct compartments, P5 and P12, composed of repeats of different lengths and periodicities. This model, developed primarily from analyses of *Leishmania* and a subset of *Trypanosoma cruzi* strains, provides a useful conceptual framework for exploring repeat architecture in the DR. In our study, the distinction between “short” and “long” repeats observed across several *Trypanosoma* species is broadly compatible with this compartmentalization.

Although the mechanisms underlying the short repetitive region changes remain unknown, it has been suggested that repetitive region sequence arrays may contain binding sites for specific transcription factors, DNA maintenance proteins or may serve as sites for initiating maxicircle replication and transcription [30]. Moreover, an intriguing conserved sequence element of ~390 bp is present in the divergent region of the *T. lainsoni* with imperfect palindromes (type I, II and III) containing a core palindromic sequence of 39 bp within the element. Palindrome structures have previously been identified in the maxicircles of other trypanosomatids [29–31,36]. These sequences have been reported in a wide range of genomes [71], most in mitochondrial DNA [72,73], as well as in chloroplasts genomes [74]. In eukaryotes, palindromic sequences have been associated with a diversity of functions. They have been linked to genetic instability, genome stability, and replication processes [75]. In addition, palindromes containing an A_5_C-element have been proposed as potential recognition sites for the binding of transcription factors or initiation of transcription [31], the exact biological function of these elements has yet to be fully explored.

In conclusion, this study presents the first description of the kDNA maxicircles in the LSRM clade. Our findings revealed that the coding region of the *T. lainsoni* maxicircle retains a conserved gene order analogous to that of other closely related trypanosomatids, with an anticipated RNA editing pattern closely resembling that of *T. cruzi.* However, *T. cruzi* requires relatively few U-insertions, supporting the idea that RNA editing may be undergoing a gradual reduction in non-salivarian *Trypanosoma* lineages. This study also supports the *T. brucei* clade as the most ancestral and shows that LSRM and aquatic trypanosomes are ancestral to non-salivarian (terrestrial) trypanosomes. Furthermore, the structure of the DR in LSRM species suggests a possible evolutionary trajectory leading to the emergence of short repeat elements. Finally, we described the presence of a palindrome containing an A_5_C within the *T. lainsoni* DR, suggesting its functional conservation across trypanosomes.

## Supporting information

S1 Fig AT skew graphs for the whole maxicircle sequence in *T. lainson*i (A) and *T. cruzi* (B). AT-rich region in *T. cruzi* Dm28c is highlighted in yellow. The window size was 100 bp.
(TIF)

S2 Fig Per-base Phred quality scores of Illumina reads mapped to the polished *T. lainsoni* maxicircle. Quality scores were calculated using Falco, based on Illumina reads previously mapped to the polished maxicircle assembly. Both forward (left) and reverse (right) reads display consistently high Phred scores across most base positions, with values exceeding Q30.
(TIF)

S3 Fig Quality and length distribution of ONT reads mapped to the *T. lainsoni* maxicircle. Summary plot generated with NanoPlot showing the relationship between read quality and read length for the Oxford Nanopore reads that mapped to the *T. lainsoni* maxicircle.
(TIF)

S4 Fig Dot plot comparisons of the maxicircle coding regions obtained using NOVOPlasty of various *Trypanosoma* species generated with YASS. The plots illustrate the sequence similarity within the LSRM clade, comparing *T. lainsoni* (Le29) with (A) *T. platydactyli,* (B) *T. scelopori*, (C) *T. lainsoni* (Ca37), and (D) *T. lainsoni* (Ca47). The axes represent sequence positions, highlighting coding regions (green lines) and regions with potential inversions or deletions (red lines). Continuous diagonal lines indicate high sequence similarity without interruptions, whereas breaks or disruptions suggest sequence variation, such as insertions, deletions, or rearrangements.
(TIF)

S5 Fig GC percentage graphs for the coding regions of the maxicircle in *T. cruzi* (A) and *T. lainsoni* (B). The x-axis represents the nucleotide position along the maxicircle coding region, and the y-axis represents the percentage of GC content. The window size used was 25 bp. Regions with GC content values exceeding the 40% threshold are indicative of RNA editing. Gray arrows: Ribosomal RNA genes. Magenta arrows: extensively edited genes. Light purple arrows: minor edited genes. Purple arrows: non-edited genes.
(TIF)

S6 Fig Dotplot comparison of maxicircle assemblies from *T. lainsoni* Le29 and Ca47 generated with NOVOPlasty. YASS alignment between the assembled contigs shows complete conservation of the coding region and a sharp divergence at the transition to the divergent region (DR). The diagonal line confirms high collinearity and sequence identity across the coding block in both isolates, while the alignment break marks the onset of the DR, consistent with the expected maxicircle architecture.
(TIF)

S7 Fig Divergent region of *T. lainsoni* maxicircle. (A) Dot plot analysis generated with YASS comparing the sequence of *T. lainsoni* maxicircle to itself, illustrating three palindromic structures: blue arrow for palindrome I, violet arrow for palindrome II, and magenta arrow for palindrome III. (B) Detailed view of the divergent region in the dot plot. (C) Presentation of palindrome sequences, their respective lengths, and their positions in the highlighted example in (B). (D) Core palindromic sequence of 39 bp within the conserved element of the divergent region.
(TIF)

S1 Table The maxicircle coding region sequences analyzed in this study. (PDF)

S2 Table ONT reads summary statistics. (PDF)

S3 Table Illumina reads summary statistics. (PDF)

S1 File Le29, Ca37, Ca47, *T. platydactyli* and *T. scelopori* maxicircle sequences. (ZIP)

S2 File Edited mRNAs. (ZIP)

S3 File BLASTn results. (XLSX)
