# Supplementary figures and images for: Comparative maxicircle analysis in Trypanosoma species from the LSRM clade highlights patterns in an underexplored lineage
Source: PLoS One. 2025 Sep 22;20(9):e0332749. doi: 10.1371/journal.pone.0332749 (PMC12453231; doi:10.1371/journal.pone.0332749)

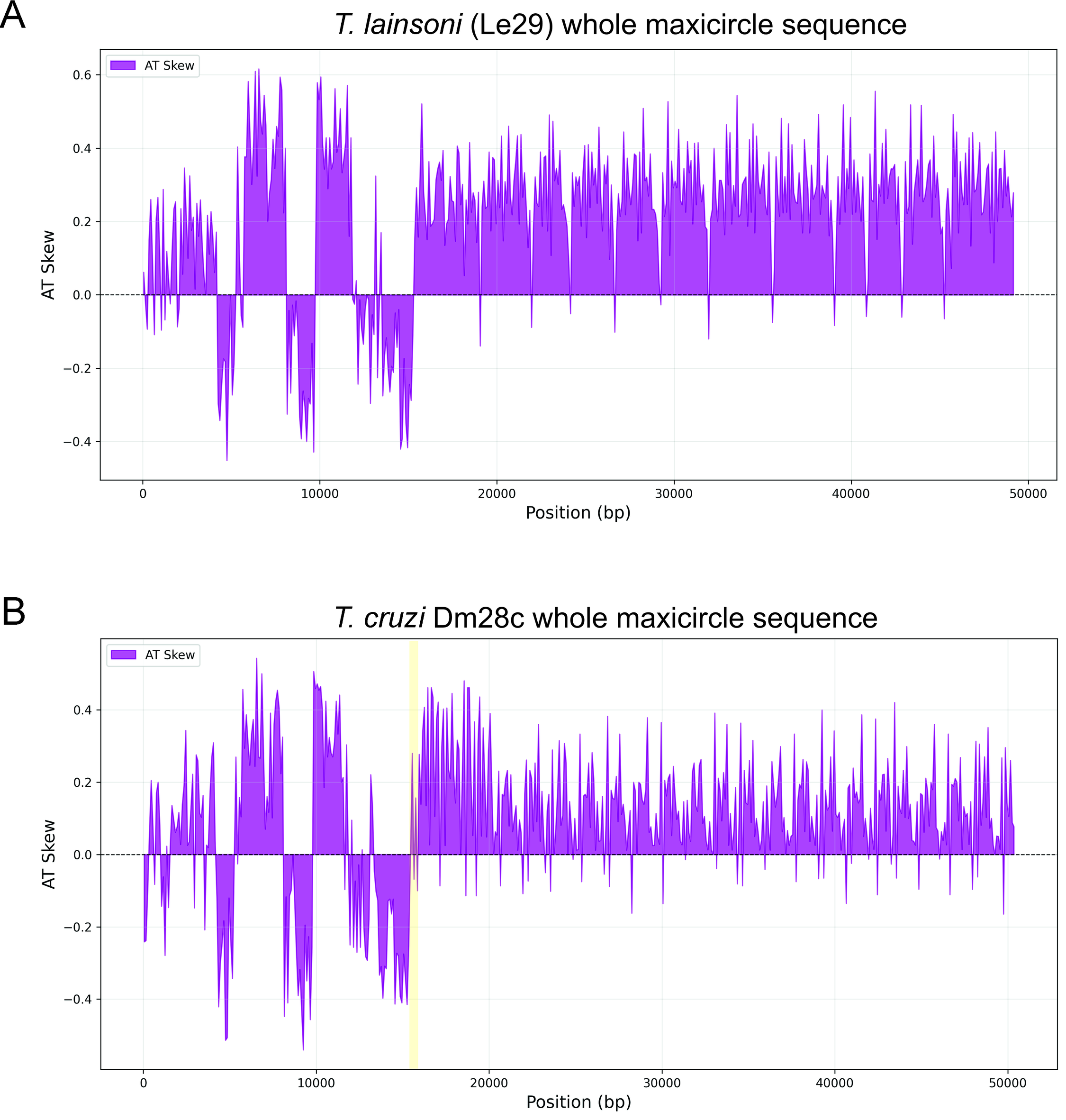

Supplement: S1 Fig — AT-rich region in T. cruzi Dm28c is highlighted in yellow. The window size was 100 bp. (TIF) [file pone.0332749.s001.tif]

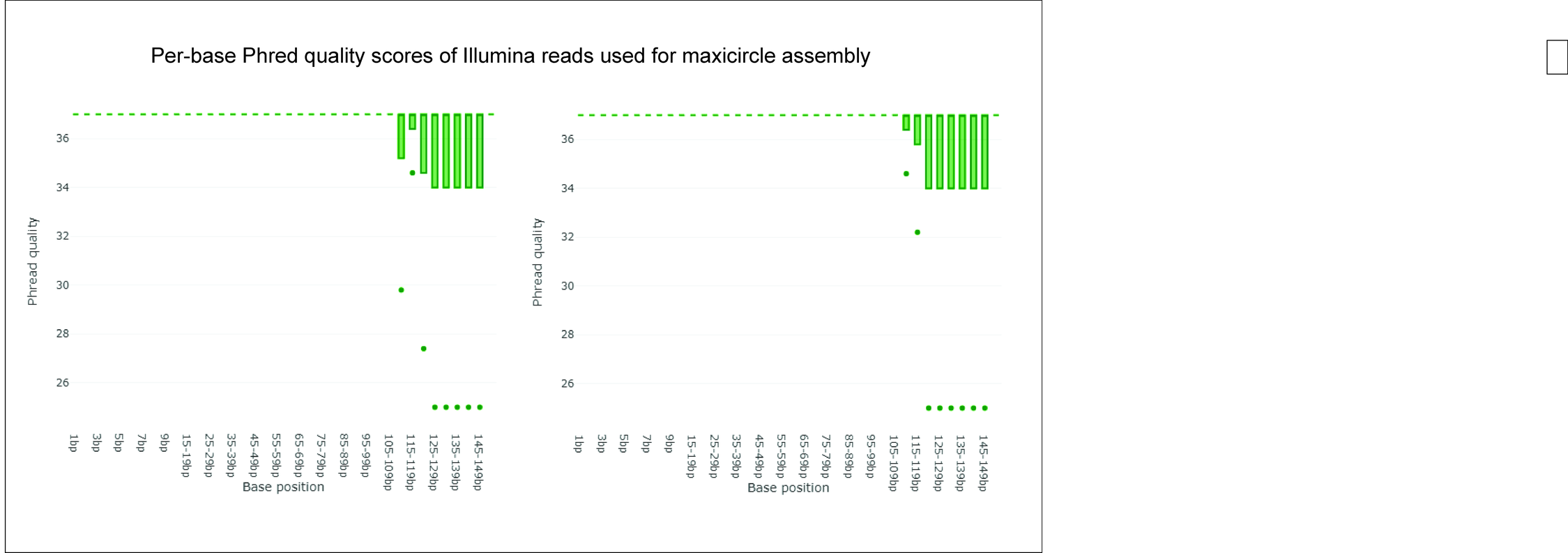

Supplement: S2 Fig — Quality scores were calculated using Falco, based on Illumina reads previously mapped to the polished maxicircle assembly. Both forward (left) and reverse (right) reads display consistently high Phred scores across most base positions, with values exceeding Q30. (TIF) [file pone.0332749.s002.tif]

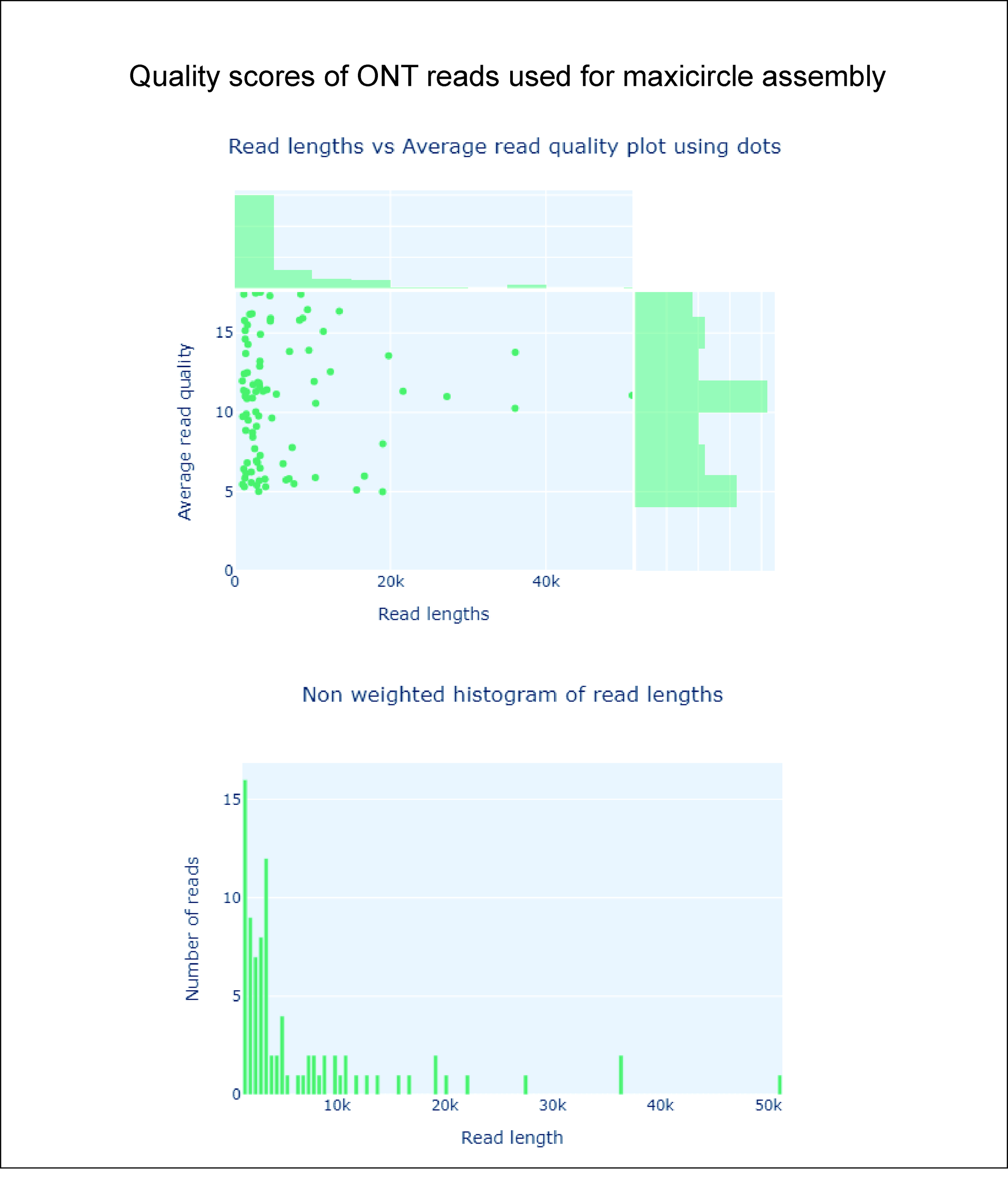

Supplement: S3 Fig — Summary plot generated with NanoPlot showing the relationship between read quality and read length for the Oxford Nanopore reads that mapped to the T. lainsoni maxicircle. (TIF) [file pone.0332749.s003.tif]

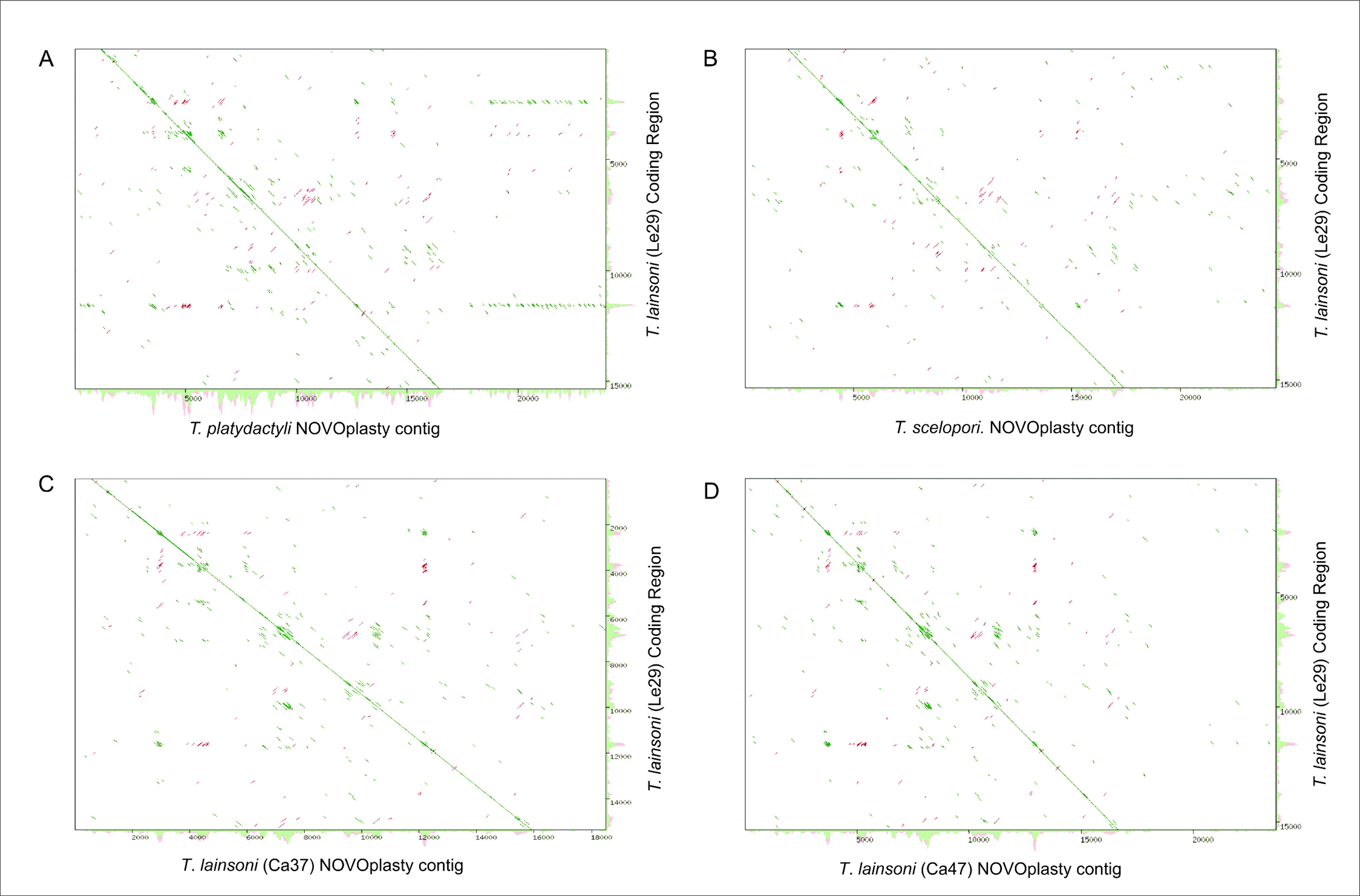

Supplement: S4 Fig — The plots illustrate the sequence similarity within the LSRM clade, comparing T. lainsoni (Le29) with (A) T. platydactyli, (B) T. scelopori, (C) T. lainsoni (Ca37), and (D) T. lainsoni (Ca47). The axes represent sequence positions, highlighting coding regions (green lines) and regions with potential inversions or deletions (red lines). Continuous diagonal lines indicate high sequence similarity without interruptions, whereas breaks or disruptions suggest sequence variation, such as insertions, deletions, or rearrangements. (TIF) [file pone.0332749.s004.tif]

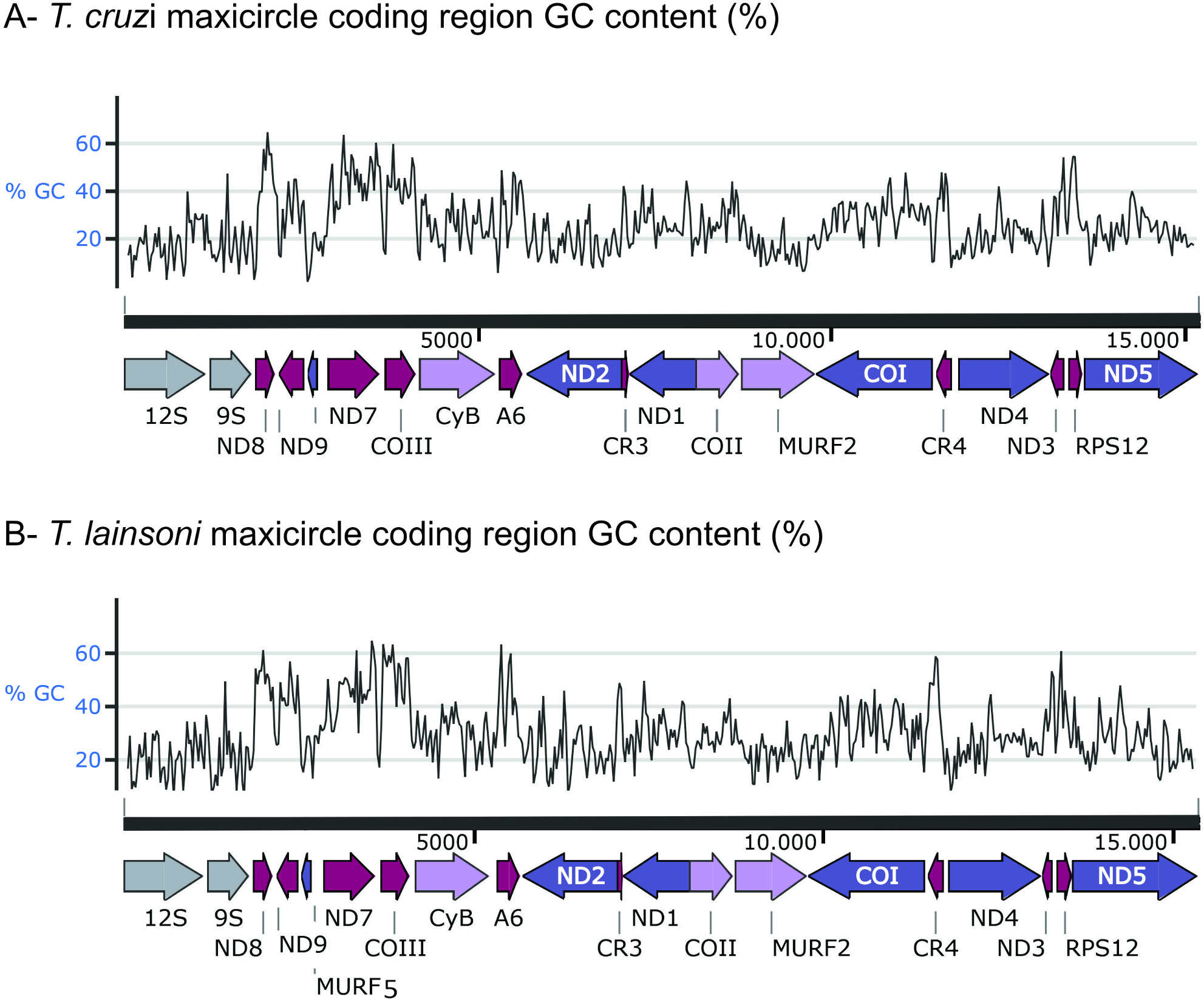

Supplement: S5 Fig — The x-axis represents the nucleotide position along the maxicircle coding region, and the y-axis represents the percentage of GC content. The window size used was 25 bp. Regions with GC content values exceeding the 40% threshold are indicative of RNA editing. Gray arrows: Ribosomal RNA genes. Magenta arrows: extensively edited genes. Light purple arrows: minor edited genes. Purple arrows: non-edited genes. (TIF) [file pone.0332749.s005.tif]

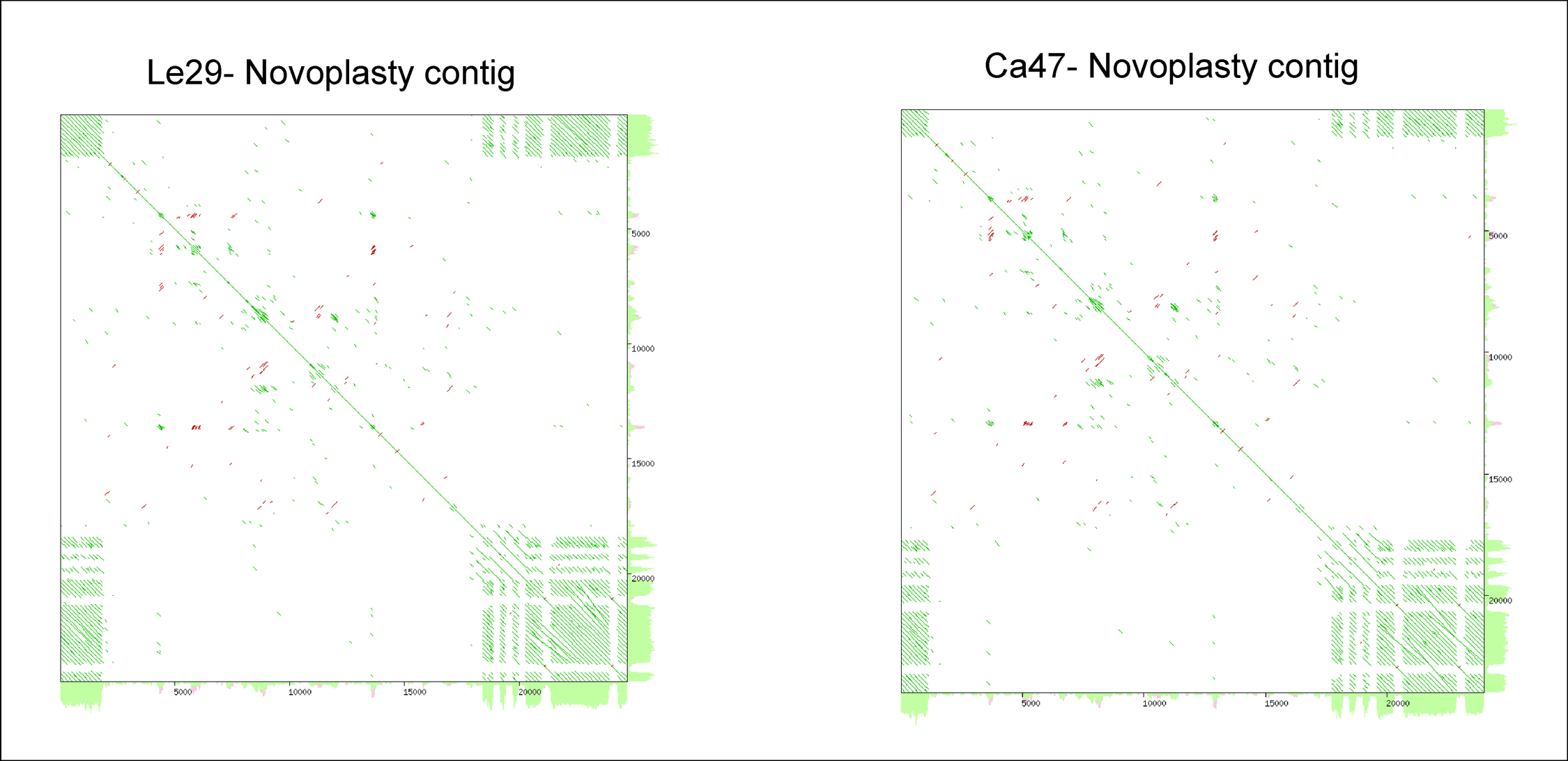

Supplement: S6 Fig — YASS alignment between the assembled contigs shows complete conservation of the coding region and a sharp divergence at the transition to the divergent region (DR). The diagonal line confirms high collinearity and sequence identity across the coding block in both isolates, while the alignment break marks the onset of the DR, consistent with the expected maxicircle architecture. (TIF) [file pone.0332749.s006.tif]

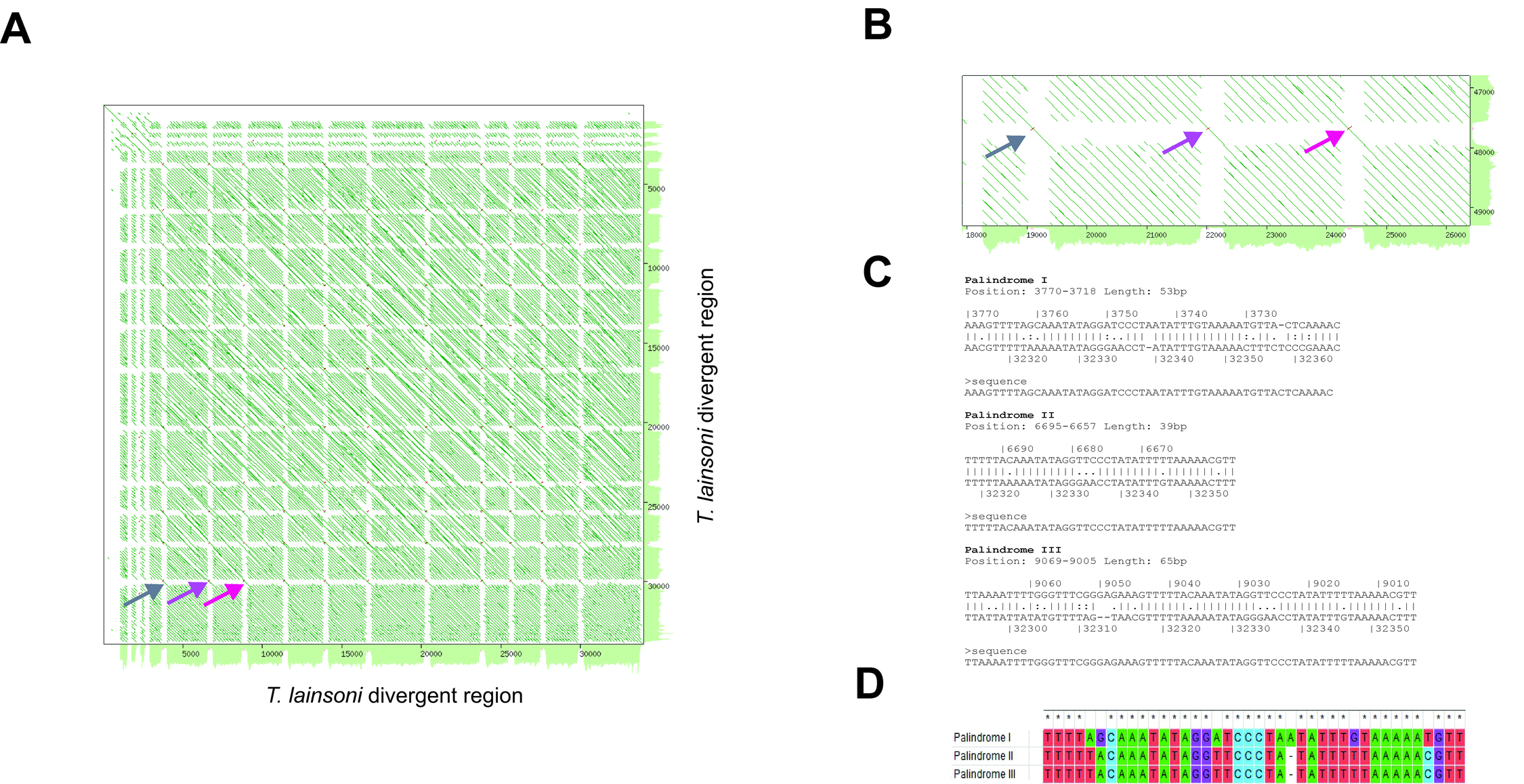

Supplement: S7 Fig — (A) Dot plot analysis generated with YASS comparing the sequence of T. lainsoni maxicircle to itself, illustrating three palindromic structures: blue arrow for palindrome I, violet arrow for palindrome II, and magenta arrow for palindrome III. (B) Detailed view of the divergent region in the dot plot. (C) Presentation of palindrome sequences, their respective lengths, and their positions in the highlighted example in (B). (D) Core palindromic sequence of 39 bp within the conserved element of the divergent region. (TIF) [file pone.0332749.s007.tif]
