# Supplementary material for: Comparative maxicircle analysis in Trypanosoma species from the LSRM clade highlights patterns in an underexplored lineage
Source: PLoS One. 2025 Sep 22;20(9):e0332749. doi: 10.1371/journal.pone.0332749 (PMC12453231; doi:10.1371/journal.pone.0332749)
Supplement: S1 Table — (PDF) [file pone.0332749.s008.pdf]

Species	Maxicircle accession code
T. cruzi- Esmeraldo	DQ343646.1
T. cruzi- CL Brener	DQ343645.1
T. cruzi- Sylvio	FJ203996.1
T. marinkellei	KC427240.1
T. conorhini	MKKU01000412.1
T. lewisi	OM000219.1
T. musculi	KT368148.1
T. copemani	MG948557.1
T. grayi	OM049542.1
T. congolense	GCA_003013265.1
T. brucei brucei	MK584625.1
L. tarentolae	NC000894.1
T. rangeli	KJ803830.1
T. theileri	GCF_002087225.1
T. brucei rhodesiense	OM049543.1
T. mega	GCA_030849715.1
T. boissoni	GCA_030849725.1
T. melophagium	GCA_022059095.1
T. equiperdum	CZPT020000280.1
T. caninum	GCA_036321205.1
