# Supplementary material for: Comparative maxicircle analysis in Trypanosoma species from the LSRM clade highlights patterns in an underexplored lineage
Source: PLoS One. 2025 Sep 22;20(9):e0332749. doi: 10.1371/journal.pone.0332749 (PMC12453231; doi:10.1371/journal.pone.0332749)
Supplement: S2 Table — (PDF) [file pone.0332749.s009.pdf]

Summary statistics

Metrics	Dataset
**number_of_reads**	88
**number_of_bases**	566475.0
**median_read_length**	3145.5
**mean_read_length**	6437.2
**read_length_stdev**	8475.3
**n50**	12300.0
**mean_qual**	9.2
**median_qual**	11.0
**longest_read_(with_Q):1**	51087 (11.1)
**longest_read_(with_Q):2**	36043 (13.8)
**longest_read_(with_Q):3**	36018 (10.3)
**longest_read_(with_Q):4**	27269 (11.0)
**longest_read_(with_Q):5**	21631 (11.3)
**highest_Q_read_(with_length):1**	17.6 (3293)
**highest_Q_read_(with_length):2**	17.5 (2690)
**highest_Q_read_(with_length):3**	17.4 (8504)
**highest_Q_read_(with_length):4**	17.4 (1161)
**highest_Q_read_(with_length):5**	17.3 (4524)
**Reads >Q10:**	51 (58.0%) 0.4Mb
**Reads >Q15:**	17 (19.3%) 0.1Mb
