# Supplementary material for: Comparative maxicircle analysis in Trypanosoma species from the LSRM clade highlights patterns in an underexplored lineage
Source: PLoS One. 2025 Sep 22;20(9):e0332749. doi: 10.1371/journal.pone.0332749 (PMC12453231; doi:10.1371/journal.pone.0332749)
Supplement: S3 Table — (PDF) [file pone.0332749.s010.pdf]

Summary statistics

Measure	Illumina R1	Illumina R2
**Filename**	illumina_R1_mapped_fastq	illumina_R2_mapped_fastq
**File type**	Conventional base calls	Conventional base calls
**Encoding**	Illumina 1.9	Illumina 1.9
**Total Sequences**	101,455	101,471
**Sequences flagged as poor quality**	0	0
**Sequence length**	50-150 bp	50-150 bp
**GC content (%)**	35%	36%
